# The F-Box Protein Fbp1 Shapes the Immunogenic Potential of *Cryptococcus neoformans*

**DOI:** 10.1128/mBio.01828-17

**Published:** 2018-01-09

**Authors:** Jorge Masso-Silva, Vanessa Espinosa, Tong-Bao Liu, Yina Wang, Chaoyang Xue, Amariliz Rivera

**Affiliations:** a Rutgers School of Graduate Studies at The Health Sciences Campus, New Jersey Medical School, Rutgers University, Newark, New Jersey, USA; b Department of Pediatrics and Center for Immunity and Inflammation, New Jersey Medical School, Rutgers University, Newark, New Jersey, USA; c Public Health Research Institute, and Department of Microbiology, Biochemistry and Molecular Genetics, New Jersey Medical School, Rutgers University, Newark, New Jersey, USA; Institut Pasteur

**Keywords:** *Cryptococcus neoformans*, T cells, immunization, monocytes

## Abstract

*Cryptococcus neoformans* is the main etiologic agent of cryptococcal meningitis and causes a significant number of deadly infections per year. Although it is well appreciated that host immune responses are crucial for defense against cryptococcosis, our understanding of factors that control the development of effective immunity to this fungus remains incomplete. In previous studies, we identified the F-box protein Fbp1 as a novel determinant of *C. neoformans* virulence. In this study, we found that the hypovirulence of the *fbp1*Δ mutant is linked to the development of a robust host immune response. Infection with the *fbp1*Δ mutant induces a rapid influx of CCR2^+^ monocytes and their differentiation into monocyte-derived dendritic cells (mo-DCs). Depletion of CCR2^+^ monocytes and their derivative mo-DCs resulted in impaired activation of a protective inflammatory response and the rapid death of mice infected with the *fbp1*Δ mutant. Mice lacking B and T cells also developed fungal meningitis and succumbed to infection with the *fbp1*Δ mutant, demonstrating that adaptive immune responses to the *fbp1*Δ mutant help to maintain the long-term survival of the host. Adaptive immune responses to the *fbp1*Δ mutant were characterized by enhanced differentiation of Th1 and Th17 CD4^+^ T cells together with diminished Th2 responses compared to the H99 parental strain. Importantly, we found that the enhanced immunogenicity of *fbp1*Δ mutant yeast cells can be harnessed to confer protection against a subsequent infection with the virulent H99 parental strain. Altogether, our findings suggest that Fbp1 functions as a novel virulence factor that shapes the immunogenicity of *C. neoformans*.

## IMPORTANCE

*Cryptococcus neoformans* is the most common cause of deadly fungal meningitis, with over 270,000 infections per year. Immune responses are critically required for the prevention of cryptococcosis, and patients with impaired immunity and low CD4^+^ T cell numbers are at high risk of developing these deadly infections. Although it is well appreciated that the development of protective immunity is shaped by the interactions of the host immune system with fungal cells, our understanding of fungal products that influence this process remains poor. In this study, we found that the activity of F-box protein 1 (Fbp1) in highly virulent *C. neoformans* clinical strain H99 shapes its immunogenicity and thus affects the development of protective immune responses in the host. The identification of this new mechanism of virulence may facilitate the future development of therapeutic interventions aimed at boosting antifungal host immunity.

## INTRODUCTION

Cryptococcal meningitis remains a significant cause of death among HIV-infected individuals throughout the world (1–3). Recent estimates indicate that 278,000 people are infected with cryptococcus every year, and that cryptococcal meningitis is responsible for 15% of AIDS-related deaths globally (3). Thus, despite significant improvements over the last decade, cryptococcosis remains an infection of global concern. Susceptibility to cryptococcosis is tightly linked to host immunity where CD4^+^ T cells play an essential role in defense (4, 5). Accordingly, a low number of CD4^+^ T cells is the primary risk factor for the development of disease (3–6). A better understanding of factors that control the activation of protective immune responses is likely to be beneficial for the future development of interventions aimed at boosting host immunity in the prevention and treatment of cryptococcosis.

Studies using mouse models of cryptococcosis have demonstrated that Th1 and Th17 CD4^+^ T cells are important in defense (4, 5, 7–9). Clinical studies similarly suggest that increased production of gamma interferon (IFN-γ), the hallmark Th1 cytokine, correlates with a better prognosis for people (8). In contrast, previous studies have shown that Th2 responses that are characterized by the production of interleukin-4 (IL-4) and IL-13 are detrimental during cryptococcosis (10–13). Thus, CD4^+^ T cell differentiation along distinct lineages has differential implications for the outcome of cryptococcosis, and can be shaped by host- and fungus-derived factors. The activation of protective, fungus-specific CD4^+^ T cell responses is critically dependent on the interaction of T cells with dendritic cells (14). Previous studies have shown that CCR2^+^ cells give rise to macrophages and dendritic cells that are essential for the development of a protective type 1 response to *Cryptococcus neoformans* (15, 16). CCR2^+^ Ly6C^hi^ monocyte-derived dendritic cells (mo-DCs) have also been shown to be important for priming protective fungus-specific CD4^+^ T cell responses in *Aspergillus* and *Blastomyces* infections and to facilitate Th1 differentiation (17–20). Thus, CCR2^+^ monocyte-derived cells play important roles in defense against a variety of fungal pathogens and act, at least in part, via the activation of protective CD4^+^ T cell responses (21, 22).

*C. neoformans* expresses a significant number of virulence factors that help fungal cells to evade host immunity (2, 23–26). Important virulence mechanisms involve the production of melanin and polysaccharide capsule, as well as the ability to grow at 37°C (thermotolerance) (23). Additional virulence factors that affect the host immune response entail the production of various enzymes, including urease and phospholipase, as well as changes in chitosan content and filamentation potential (13, 26–29). In previous studies, we identified the F-box protein Fbp1 as a novel virulence factor in highly virulent *C. neoformans* strain H99 (30, 31). Fbp1 functions as a subunit of the SCF^Fbp1^ E3 ligase complex, a key component of the ubiquitin-mediated proteolytic pathway that targets specific proteins for ubiquitination and subsequent degradation (31, 32). We found that mice infected with the *fbp1*Δ mutant (H99 with Fbp1 deleted) survived long term, and maintained a persistent pulmonary fungal burden throughout infection without meningitis development (30). The hypovirulent phenotype of the *fbp1*Δ mutant did not involve changes in capsule size, melanin production, or thermotolerance, suggesting that the Fbp1-regulated mechanism of virulence operates independently of these virulence factors.

In this study, we set out to define the underlying mechanisms that contribute to the *in vivo* hypovirulent phenotype of the *fbp1*Δ mutant. We found that infection with the *fbp1*Δ mutant induced a robust inflammatory response and enhanced the activation of innate and adaptive immune responses compared to the parental H99 strain. Infection with the *fbp1*Δ mutant promoted the enhanced activation of Th1 and Th17 differentiation together with diminished Th2 responses. Long-term protection from *fbp1*Δ mutant yeast infection was dependent on the activation of adaptive immune responses since lymphocyte-deficient (RAG^−/−^) mice were not able to control infection with the *fbp1*Δ mutant and succumbed to infection. The activation of protective immunity to the *fbp1*Δ mutant was also facilitated by increased innate responses. Infection with the *fbp1*Δ mutant induced a more robust recruitment of CCR2^+^ Ly6C^+^ monocytes and their maturation into mo-DCs compared with H99. Depletion of CCR2^+^ cells in CCR2 depleter (CCR2-DTR) mice resulted in the abrogation of protective immunity and rapid death of *fbp1*Δ mutant yeast*-*infected mice. Importantly, vaccination with the *fbp1*Δ mutant resulted in the induction of immune responses that protected the host against a lethal challenge with the parental H99 strain. Altogether, our results suggest that Fbp1 acts as a virulence factor that shapes the immunogenicity of *C. neoformans*. A disruption of this Fbp1-controlled pathway is sufficient to induce robust innate and adaptive immune responses that protect the host from infection and can be harnessed in vaccination strategies. Overall, our study uncovered a novel point of host-pathogen interaction that shapes the immunogenicity of *C. neoformans*.

## RESULTS

### Deletion of Fbp1 does not affect the expression of several major virulence factors in *C. neoformans.*

**FIG 1:**
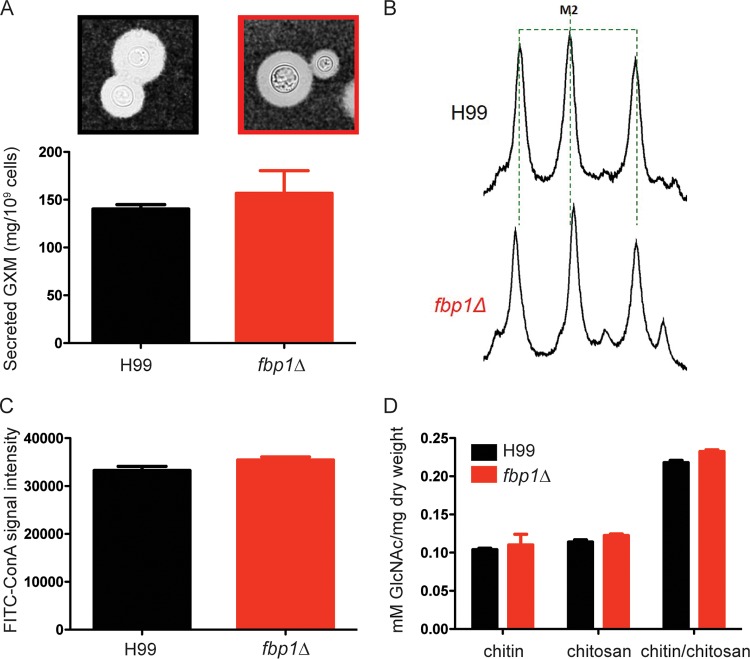
Deletion of Fbp1 does not affect the expression of several major virulence factors in *C. neoformans*. (A) GXM secretion was measured after H99 and the *fbp1*Δ mutant were grown on capsule-inducing MM for 3 days. A typical yeast cell and the capsule of each strain following Indian ink staining are shown at the top. (B) One-dimensional proton NMR spectroscopic detection of the GXM structure produced by H99 and *fbp1*Δ mutant cells. The spectrum peaks indicate the M2 mannosyl triad structure reporter groups. (C) Cultures of H99 and *fbp1*Δ mutant cells were incubated with ConA-FITC, and the fluorescence was quantified by flow cytometry. The bar graph represents the average signal intensity. (D) Chitin and chitosan production was compared in H99 and *fbp1*Δ mutant cells grown on YPD overnight as described in Materials and Methods. Differences in all of the graphs were not statistically significant, as examined by the Mann-Whitney test.

In previous studies, we determined that deletion of Fbp1 from *C. neoformans* strain H99 resulted in hypovirulence *in vivo* without affecting the production of primary virulence factors such as melanin, capsule, and thermotolerance *in vitro* (32). To further examine potential effects on known virulence factors, we performed a detailed characterization of the secretion of the primary capsule polysaccharide glucuronoxylomannan (GXM) by the *fbp1*Δ mutant and the parental strain and determined that both strains secrete comparable amounts (Fig. 1A). We also tested the capsule composition in detail and found no differences in size (Fig. 1A) or structure (Fig. 1B). Previous studies have suggested that changes in mannoproteins, chitin, or chitosan can impact the virulence of *C. neoformans* (13, 24, 27, 33). Therefore, we examined whether the *fbp1*Δ mutant had changes in these components compared to parental strain H99. We employed a previously published concanavalin A (ConA)-fluorescein isothiocyanate (FITC) binding assay (33) to measure surface mannoproteins expressed by the *fbp1*Δ mutant and H99 and did not detect any significant differences (Fig. 1C). Similarly, we found that there were no significant differences in the amount of chitin or chitosan (34) expressed by the *fbp1*Δ mutant and H99 (Fig. 1D). In aggregate, these results together with our previously published analyses (30) suggest that the attenuated virulence *in vivo* upon infection with the *fbp1*Δ mutant cannot be explained by measurable changes in the production of these important virulence factors. We, therefore, sought to examine whether infection with the *fbp1*Δ mutant impacts the development of host immunity compared to parental strain H99 and to define host factors that might confer protection from infection with the *fbp1*Δ mutant.

### Infection with the *fbp1*Δ mutant induces a robust inflammatory response in the host.

**FIG 2:**
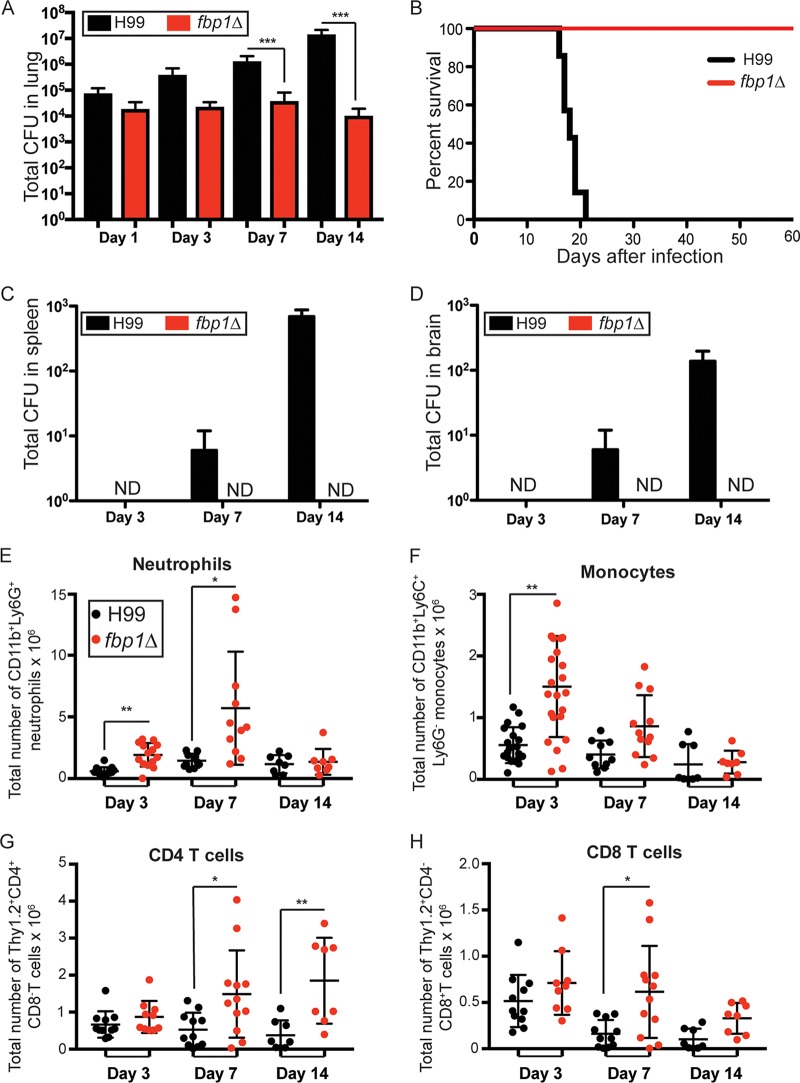
Mice infected with the *fbp1*Δ mutant display enhanced inflammatory responses and survival. C57BL/6J mice were infected i.n. with 10^5^ wild-type H99 cells (black symbols) or 10^6^ *fbp1*Δ mutant cells (red symbols). (A) Total fungal burden in the lungs (CFU). The data shown are the mean ± the standard error of the mean of 5 to 12 mice per group per time point and are cumulative from two independent experiments. (B) Survival rates of C57BL/6J mice after infection. The data shown in each graph are for eight mice per group and are cumulative from two independent experiments. (C and D) Fungal burdens determined by CFU recovery from the spleen (C) and brain (D). The data shown are the mean ± the standard error of the mean of 5 to 12 mice per group per time point and are cumulative from two independent experiments. (E to H) Cellular infiltration of the lungs was analyzed by flow cytometry. Each symbol represents one mouse, and the data are cumulative from two independent experiments. Bars represent the mean ± the standard error of the mean. Each cell population was identified as CD45^+^ 4',6-diamidino-2-phenylindole (DAPI)-negative live leukocytes, neutrophils were gated as CD11b^+^ Ly6C^+^ Ly6G^+^ (E), monocytes were gated as CD11b^+^ Ly6C^+^ Ly6G^−^ (F), CD4^+^ T cells were gated as Thy1.2^+^ CD11b^−^ CD4^+^ CD8^−^, and CD8 T cells were gated as Thy1.2^+^ CD11b^−^ CD4^−^ CD8^+^. Statistical analysis of survival curves was done by log rank (Mantel-Cox) test. ***, *P* ≤ 0.001. Statistical analysis of all of the other graphs shown was done with the Kruskal-Wallis nonparametric test for multiple comparisons. ND, not detected. *, *P* ≤ 0.05; **, *P* ≤ 0.01; ***, *P* ≤ 0.001 (for compared groups that showed statistically significant differences).

In our previous study, we employed an infectious dose of 10^5^ H99 cells or 10^5^ *fbp1*Δ mutant yeast cells in mice of the A/Jcr background, and found that these mice survived infection with the *fbp1*Δ mutant for over 60 days (30). We observed that although the initial inoculum was the same, H99 was able to replicate in the lungs and disseminate to the brain, and spleen, while the *fbp1*Δ mutant remained in the lungs (30). In this study, we wanted to determine whether increasing the infection dose of the *fbp1*Δ mutant would overcome this attenuated phenotype. We also wanted to examine whether attenuation of the *fbp1*Δ mutant was maintained in mice of a different genetic background since previous studies reported substantial intrinsic differences among mouse strains in terms of their susceptibility to cryptococcosis (7, 35–41). We chose to test mice in the C57BL/6J (B6) background since this is a commonly studied strain for analyses of host immunity, and there are a significant number of immune gene-deficient mice in this background. Infection of C57BL/6J mice with 10^5^ H99 cells or 10^6^ cells of the *fbp1*Δ mutant resulted in equal seeding of yeast cells in the lungs at 24 h after infection (Fig. 2A). All C57BL/6J mice infected with H99 succumbed to the infection by 20 days after inoculation, while *fbp1*Δ mutant-infected mice survived for over 50 days (Fig. 2B). We observed a similar survival pattern in A/Jcr mice infected with 10^6^ *fbp1*Δ mutant cells (see Fig. S1A in the supplemental material). Therefore, increasing the infection dose or changing the host genetic background did not overcome the attenuation of the *fbp1*Δ mutant. We observed that the *fbp1*Δ mutant fungal burden remained fairly constant in the lungs of infected mice during our observation period (Fig. 2A). In contrast, H99 continued to grow, and by day 14 after infection, the pulmonary fungal burden of H99 had increased to several logs higher than the initial number of yeast cells seeded into the lungs at 24 h after inoculation (Fig. 2A). H99 was also able to quickly escape from the lungs, and viable CFU were recovered from the spleen and brain as early as day 7 after infection (Fig. 2C and D). In contrast, the *fbp1*Δ mutant remained in the lungs, and we did not detect viable yeast cells from the spleens or brains of infected mice (Fig. 2A, C, and D). Thus, H99 is able to rapidly overcome the host, and disseminates to extrapulmonary sites while the *fbp1*Δ mutant remains in the lungs. On the basis of our observations thus far, we hypothesized that changes in the host immune response might be central to the pulmonary containment of the *fbp1*Δ mutant and the long-term survival of mice infected with the *fbp1*Δ mutant compared to H99. Indeed, several studies have documented the critical importance of host immunity as a determinant of the outcome of infection with *C. neoformans* mutants (27–29, 42, 43). As a first step in the analysis of host responses, we examined the recruitment of immune cells to the lungs by flow cytometric analysis. We observed that infection with the *fbp1*Δ mutant induced a significant increase in the number of innate and adaptive immune cells that were recruited to the lungs (Fig. 2E to H). We observed increased numbers of neutrophils and monocytes (Fig. 2E and F), as well as increased numbers of pulmonary CD4^+^ and CD8^+^ T cells (Fig. 2G and H). In aggregate, these observations suggest that host immune cells are more robustly recruited to the lungs of mice infected with yeast cells lacking Fbp1 compared to parental strain H99.

10.1128/mBio.01828-17.1 FIG S1  (A) A/Jcr mice were infected i.n. with 10^5^ H99 cells or 10^6^ *fbp1*Δ mutant cells. Survival rates of A/J mice after infection are shown. The data are for eight mice per group and are cumulative from two independent experiments. (B to G) A/Jcr mice were vaccinated i.n. with HK *fbp1*Δ mutant cells on days −32 and −7. On day 0, vaccinated and unvaccinated controls were infected with 10^4^ virulent H99 cells. (B) Total numbers of CD4^+^ T cells recovered from the BALF of naive mice (black symbols) and HK *fbp1*Δ mutant yeast-vaccinated C57BL/6J mice that survived for 72 days after an H99 challenge (red symbols). Each symbol represents one mouse. (C) Representative FACS plot of intracellular cytokine production by CD4^+^ T cells recovered from the BALF of vaccinated C57BL/6J mice 72 days after an H99 challenge. (D) Percentages of IFN-γ- and IL-17A-producing CD4^+^ T cells in vaccinated mice as analyzed by FACS. Each symbol represents one mouse. The data shown are cumulative from two independent experiments. (E to G) CD4^+^ T cells were isolated from the lung-draining lymph nodes of *fbp1*Δ mutant-vaccinated mice that survived an H99 challenge for 72 days. Cytokine secretion in the presence or absence of *C. neoformans* (Cn) antigens (Ag) was examined by ELISA as described in Materials and Methods. The data shown are cumulative from three independent experiments with four or five mice per group and are depicted as the mean ± the standard error of the mean. **, *P* ≤ 0.01 (determined by log rank [Mantel-Cox] test [A and B] or Mann-Whitney test [E and G to I]). Download FIG S1, TIF file, 1.6 MB. Copyright © 2018 Masso-Silva et al. 2018 Masso-Silva et al. This content is distributed under the terms of the Creative Commons Attribution 4.0 International license.

### Altered differentiation of CD4^+^ T cells in response to infection with the *fbp1*Δ mutant.

**FIG 3:**
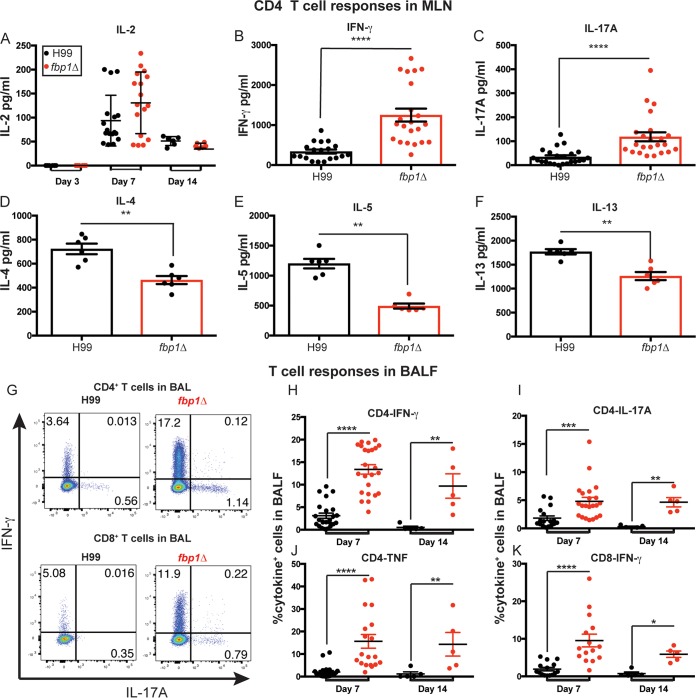
Enhanced Th1 and Th17 CD4^+^ T cell responses of mice infected with the *fbp1*Δ mutant. (A to F) *Cryptococcus*-specific CD4^+^ T cell responses were examined in lung-draining lymph nodes (MLNs) after infection with H99 (black symbols) or the *fbp1*Δ mutant (red symbols). CD4^+^ T cells were purified from the MLNs of infected mice and stimulated with APCs in the presence or absence of *C. neoformans* sonicated antigens. The data shown are for cytokines secreted into the supernatant after 72 h of *in vitro* restimulation. No cytokines were detected in samples cultured without *C. neoformans* antigen. Each symbol represents one mouse. The data shown are cumulative from three independent experiments with four or five mice per group. (A) IL-2 production on days 3, 7, and 14 after infection. (B to F) Cytokine production was measured by ELISA at the response peak on day 7. (B to F) Production of IFN-γ (B), IL-17A (C), IL-4 (D), IL-5 (E), and IL-13 (F). (G) Representative fluorescence-activated cell sorter (FACS) plots of CD4^+^ (top) and CD8^+^ (bottom) T cells in BALF. (H to K) Cytokine expression analyzed by ICCS. Each symbol represents one mouse. Data are cumulative from four independent experiments with five mice per group. (H to J) Plots of cytokine production in CD4^+^ T cells gated as Thy1.2^+^ CD4^+^ CD8^−^ T cells. The frequencies of IFN-γ (H)-, IL-17A (I)-, and TNF (J)-producing CD4^+^ T cells in BALF were analyzed as shown in panel G. (K) Frequency of IFN-γ-producing Thy1.2^+^ CD4^−^ CD8^+^ T cells. Statistical analysis of data shown in panels A and H to K was done with the Kruskal-Wallis nonparametric test for multiple comparisons. Graphs B to F were analyzed by Mann-Whitney test. *, *P* ≤ 0.05; **, *P* ≤ 0.01; ***, *P* ≤ 0.001; ****, *P* ≤ 0.0001 (for compared groups that showed statistically significant differences).

Given the importance of CD4^+^ T cells in defense against cryptococcosis (4, 44, 45), we next examined the differentiation of *Cryptococcus*-specific CD4^+^ T cells in mice infected with Fbp1-deficient or -sufficient yeast. To this end, we examined the cytokine profile of CD4^+^ T cells recovered from the airways and lung-draining mediastinal lymph nodes (MLNs) of mice infected with the *fbp1*Δ mutant compared to H99. We employed our previously developed methods to examine CD4^+^ T cell responses (19, 46, 47). Significant expansion of cytokine-producing CD4^+^ T cells in the MLNs was detected on days 7 and 14 after infection (Fig. 3A). Infections with H99 and the *fbp1*Δ mutant induced comparable CD4^+^ T cell activation in the MLNs, as examined by IL-2 production after *ex vivo* restimulation (Fig. 3A). The peak production of cytokines by polarized CD4^+^ T cells in the MLNs occurred on day 7 (Fig. 3A to F). We observed that infection with the *fbp1*Δ mutant induced enhanced differentiation of IFN-γ-secreting Th1 cells (Fig. 3B) and IL-17A-secreting Th17 CD4^+^ T cells (Fig. 3C). Increased differentiation of Th1 and Th17 cells after *fbp1*Δ mutant yeast infection was accompanied by decreased differentiation of IL-4 (Fig. 3D)-, IL-5 (Fig. 3E)-, and IL-13 (Fig. 3F)-producing Th2 cells compared to H99 infection. Thus, infection with the *fbp1*Δ mutant seems to skew CD4^+^ T cell polarization toward Th1 and Th17 responses and diminished Th2 differentiation. On days 7 and 14, mice infected with the *fbp1*Δ mutant also displayed enhanced frequencies of IFN-γ^+^, IL-17A^+^, and TNF^+^ CD4^+^ T cells that infiltrated the airways compared to mice infected with H99 (Fig. 3G to J). Increased frequencies of IFN-γ-producing CD8^+^ T cells were also present in the airways of *fbp1*Δ mutant yeast*-*infected mice (Fig. 3G and K). Collectively, these findings indicate that infection with the *fbp1*Δ mutant induces enhanced recruitment of innate and adaptive immune cells, as well as increased induction of Th1 and Th17 responses, and lower Th2 CD4^+^ T cell responses. Previous studies have shown that Th2 responses to *C. neoformans* are detrimental while increased Th1 responses are protective (16, 35, 37, 38, 40, 41, 43–45, 48–50). Therefore, the observed long-term survival of *fbp1*Δ mutant yeast*-*infected mice might be explained, at least in part, by enhanced induction of protective host immune responses that help restrain the *fbp1*Δ mutant.

### Lymphocytes are required to maintain the long-term survival of mice infected with *fbp1*Δ mutant *C. neoformans*.

**FIG 4:**
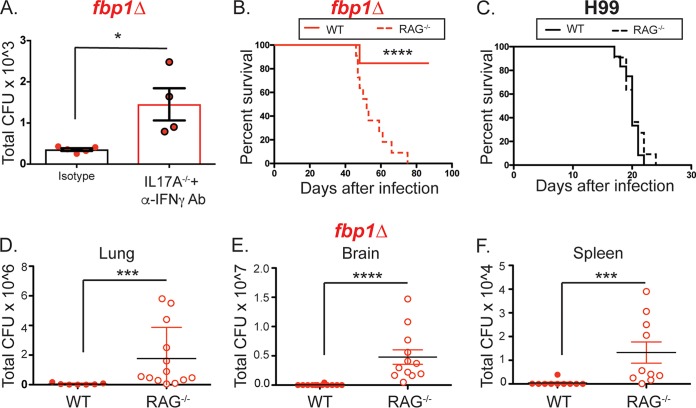
Lymphocytes are required for long-term protection against infection with the *fbp1*Δ mutant. (A) Fungal burdens on day 15 after *fbp1*Δ mutant infection in wild-type (WT) mice treated with control antibodies (black bar) and IL-17A^−/−^ mice treated with anti-IFN-γ neutralizing antibodies (Ab) (red bar). (B and C) Lymphocyte-deficient mice (RAG^−/−^, dashed lines) and control mice (solid lines) were infected i.n. with 10^5^ H99 cells (black lines) or 10^6^ *fbp1*Δ mutant cells (red lines). Survival data shown are cumulative from two independent experiments with five to seven mice per group. (B) Survival rates of RAG^−/−^ and wild-type control mice after infection with the *fbp1*Δ mutant. (C) Survival rates of RAG^−/−^ and wild-type control mice after infection with H99. (D-F) Numbers of CFU in the lungs (D), brains (E), and spleens (F) of RAG^−/−^ and wild-type mice infected with the *fbp1*Δ mutant. Each symbol represents one mouse. The data shown are cumulative from two independent experiments. *, *P* ≤ 0.05; ***, *P* ≤ 0.001; ****, *P* ≤ 0.0001 (determined by log rank [Mantel-Cox] test [B and C] or Mann-Whitney test [A and D to F]).

Our findings so far indicate that infection with the *fbp1*Δ mutant induces an enhanced activation of immune responses, including increased differentiation of Th1 and Th17 cells as well as increased production of IFN-γ by CD8^+^ T cells (Fig. 3). Therefore, enhanced adaptive immunity to infection with the *fbp1*Δ mutant might be an important protective component of the host response. To begin addressing the potential contributions of lymphocytes to the host defense upon infection with the *fbp1*Δ mutant, we treated IL-17A^−/−^ mice with neutralizing antibodies to IFN-γ to target the main effector cytokines of Th1 and Th17 cells. We observed that IFN-γ neutralization in IL-17A^−/−^ mice resulted in greater pulmonary *fbp1*Δ mutant fungal burdens than in wild-type animals treated with isotype control antibodies (Fig. 4A). Thus, increased production of IFN-γ and IL-17A in *fbp1*Δ mutant yeast*-*infected mice contributes to pulmonary control of infection. To further test the importance of lymphocytes in this response, we infected RAG^−/−^ mice, which lack mature B and T cells, with the *fbp1*Δ mutant and the parental H99 strain. Remarkably, 100% of mice lacking lymphocytes succumbed to infection with the *fbp1*Δ mutant while 85% of normal control mice survived for over 76 days after infection (Fig. 4B). In contrast, mice infected with the parental H99 strain died within 21 days after infection whether they had an intact lymphoid compartment or not (Fig. 4C). Analysis of fungal burdens in *fbp1*Δ mutant yeast*-*infected RAG^−/−^ mice at the time of euthanasia showed an increased fungal burden in the lungs, as well as dissemination of *fbp1*Δ mutant cells to the spleen and brain (Fig. 4D to F). Altogether, these findings indicate that an intact lymphoid compartment is required for the protection and long-term survival of mice infected with the *fbp1*Δ mutant.

### Enhanced recruitment and maturation of CCR2^+^ Ly6C^+^ monocytes in mice infected with Fbp1-deficient yeast.

**FIG 5:**
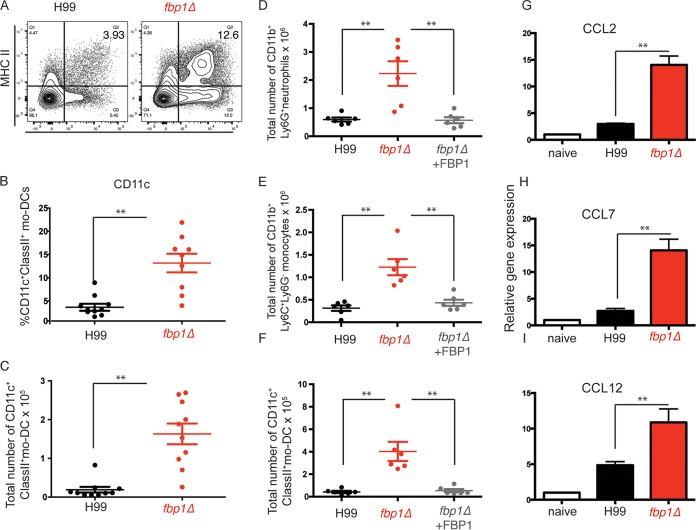
Effective maturation of CCR2^+^ Ly6C^+^ monocytes into mo-DCs after *fbp1*Δ mutant yeast infection. Differentiation of mo-DCs was analyzed on day 3 after i.n. infection with H99 (black bars) or the *fbp1*Δ mutant (red bars). The data shown are cumulative from two independent experiments with four or five mice per group and are depicted as the mean ± the standard error of the mean. (A) Representative FACS profile of Ly6C^+^ monocyte maturation into mo-DCs (defined as CD45^+^ CD11b^+^ Ly6C^hi^ Ly6G-CD11c^+^ class II^+^) in H99- or *fbp1*Δ mutant-infected mice. MHC, major histocompatibility complex. (B) Percentages of mo-DCs (CD11c^+^ class II^+^) in monocyte gate (CD45^+^ CD11b^+^ Ly6C^hi^ Ly6G^−^) in mice infected with H99 (black bar) or the *fbp1*Δ mutant (red). (C) Total numbers of mo-DCs recruited to the lungs. (D to F) Total numbers of recruited neutrophils (D), monocytes (E), and mo-DCs (F) on day 3 after infection with H99 (black symbols), the *fbp1*Δ mutant (red symbols), or the complemented strain (*fbp1*Δ *FBP1*) (gray symbols). (G to I) Chemokine expression in lung tissue was analyzed by qRT-PCR. The expression of each CCR2 ligand was examined with TaqMan probes. Differential gene expression relative to GAPDH was calculated by the ΔΔ*CT* method. **, *P* ≤ 0.01 (determined by Mann-Whitney test).

Although 100% of *fbp1*Δ mutant yeast-infected RAG^−/−^ mice succumbed to infection, there was an average 30-day delay of death compared to H99-infected mice (Fig. 4B and C). This observation suggests that immune cells other than lymphocytes can also help restrain infection with the *fbp1*Δ mutant. Our analysis of immune cell infiltration to the lungs of *fbp1*Δ mutant yeast*-*infected mice showed significant increases in the number of recruited monocytes (Fig. 2F). In previous studies, we observed that CCR2^+^ Ly6C^+^ monocytes are important precursors of mo-DCs that orchestrate the development of Th1 CD4^+^ T cell responses to pulmonary fungal infection (17, 19). Moreover, CCR2^+^ monocytes have been previously shown to be important innate cells that contribute to defense against infection with *C. neoformans* (15, 16, 51–55). In addition, studies of pulmonary *Blastomyces dermatitidis* infection have demonstrated that inhibition of CCR2^+^ Ly6C^+^ influx is a mechanism of virulence employed by other fungi (14, 18, 56). Importantly, blockade of CCR2^+^ Ly6C^+^ influx by *B. dermatitidis* results in impaired immunity, while robust recruitment correlates with induction of protective immunity (18, 56). We thus hypothesized that the Fbp1-regulated mechanism of virulence in *C. neoformans* might similarly affect the recruitment of Ly6C^+^ monocytes. To test this hypothesis, we examined the differentiation of monocytes into mo-DCs on day 3 after *C. neoformans* infection with H99 or the *fbp1*Δ mutant. Infection with Fbp1-deficient yeast resulted in a significantly increased influx of monocytes (Fig. 2F), as well as their maturation into CD11c^+^ class II^+^ mo-DCs (Fig. 5A to C), as examined by the percentage of CD11c^+^ class II^+^ cells among monocytes (Fig. 5A and B) and by the total number of mo-DCs recruited to the lungs (Fig. 5C). Enhanced influx of innate cells was not evident in mice infected with H99 or the *FBP1*-complemented mutant strain (*fbp1*Δ *FBP1*) (Fig. 5D to F). Thus, expression of Fbp1 in *C. neoformans* correlates with limited recruitment of inflammatory monocytes and mo-DCs. Increased influx of Ly6C^+^ monocytes in *fbp1*Δ mutant*-*infected mice correlated with higher production of CCR2 ligands CCL2, CCL7, and CCL12 (Fig. 5G to I). These observations suggest that increased recruitment of CCR2^+^ Ly6C^+^ monocytes and their differentiation into mo-DCs are a potentially important innate mechanism of protection in mice infected with the *fbp1*Δ mutant.

### CCR2^+^ cells are required for the survival of *fbp1*Δ mutant yeast*-*infected mice.

**FIG 6:**
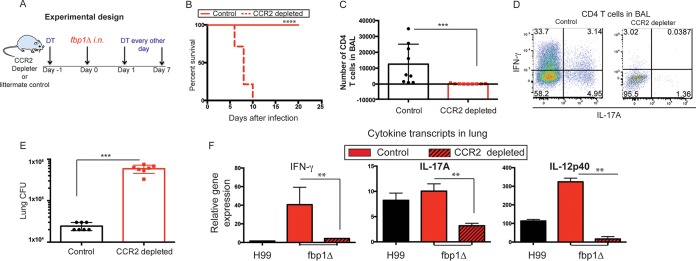
Depletion of CCR2^+^ cells impairs CD4^+^ T cell activation and leads to the death of *fbp1*Δ mutant yeast-infected mice. CCR2-DTR mice and control (DTR-negative) littermates were treated with 250 ng of DT intraperitoneally before and after infection with 10^6^ *fbp1*Δ mutant cells as illustrated in panel A. (B) Survival curve of *fbp1*Δ mutant yeast*-*infected, CCR2-depleted mice (dashed red line) and control littermates (solid red line). The data shown are cumulative from three independent experiments with four or five mice per group. ****, *P* ≤ 0.0001 (determined by log rank [Mantel-Cox] test). (C) Total number of CD4^+^ T cells recovered from the BALF of *fbp1*Δ mutant*-*infected, CCR2-depleted mice (red symbols) and control littermates (black symbols) on day 6 after infection. Each symbol represents one mouse. The data shown are cumulative from two experiments with four or five mice per group ***, *P* ≤ 0.001 (determined by Mann-Whitney test). (D) Representative FACS profile of cytokine production by CD4^+^ T cells recovered from the BALF of *fbp1*Δ mutant*-*infected, CCR2-depleted mice or control littermates. Plots are gated on Thy1.2^+^ CD4^+^ CD8^−^ lymphocytes in BALF. (E) CFU counts in lung tissue of *fbp1*Δ mutant*-*infected, CCR2-depleted mice (red symbols) and control littermates (black symbols) on day 6 after infection. Data are cumulative from two independent experiments with three or four mice per group. ***, *P* ≤ 0.001 (determined by Mann-Whitney test). (F) Cytokine gene transcription in lung tissue was examined on day 6 after infection with the *fbp1*Δ mutant in CCR2-depleted mice (red bars) and control littermates (red striped bars). Control C57BL/6J mice infected with H99 were also analyzed as a control population (black bars). Differential gene expression relative to GAPDH was examined by qRT-PCR with cytokine-specific TaqMan probes and calculated by the ΔΔ*CT* method. The data shown are cumulative from two independent experiments with four mice per group and are depicted as mean ± the standard error of the mean. **, *P* ≤ 0.01 (determined by Mann-Whitney test).

To test the potential contributions of CCR2^+^ monocytes and their derivative cells to host-mediated defense against infection with the *fbp1*Δ mutant, we employed the CCR2-DTR mouse strain, which permits the temporal removal of CCR2^+^ cells upon diphtheria toxin (DT) administration (17, 57). CCR2-DTR and control littermates were infected with the *fbp1*Δ mutant and treated with DT as depicted in Fig. 6A. Removal of CCR2^+^ cells resulted in the rapid death of mice (Fig. 6B) compared to control mice. In the absence of CCR2^+^ cells, there was minimal recruitment of CD4^+^ T cells to the airways (Fig. 6C), and the few cells that infiltrated the airways failed to differentiate into IFN-γ- or IL-17A-producing cells (Fig. 6D). Depletion of CCR2^+^ cells also resulted in a failure to induce the activation of *Cryptococcus*-specific CD4^+^ T cell responses in the MLNs (data not shown). Removal of CCR2^+^ cells and impaired CD4^+^ T cell responses were also accompanied by a failure to restrain fungal growth in the lungs (Fig. 6E). The expression of protective cytokines in the lungs was also significantly diminished in *fbp1*Δ mutant yeast*-*infected mice that were depleted of CCR2^+^ cells (Fig. 6F). Although CCR2 is expressed by several immune cell populations, including subsets of NK cells and CD8^+^ T cells, the primary cell population targeted in this model are CCR2^+^ monocytes and their derivative cells (17, 19, 57, 58). Thus, the enhanced susceptibility of CCR2-depleted mice to infection with the *fbp1*Δ mutant (Fig. 6) is most likely due to the contributions of CCR2^+^ monocytes and mo-DCs. Altogether, these observations suggest that increased influx of CCR2^+^ monocytes and their differentiation into mo-DCs upon infection with the *fbp1*Δ mutant (Fig. 2F and 5) are helpful in the containment of infection. Hence, in the absence of CCR2^+^ cells, in particular, CCR2^+^ monocytes and mo-DCs, the *fbp1*Δ mutant is a virulent strain.

### Vaccination with the *fbp1*Δ mutant confers protection from infection with the virulent H99 strain.

**FIG 7:**
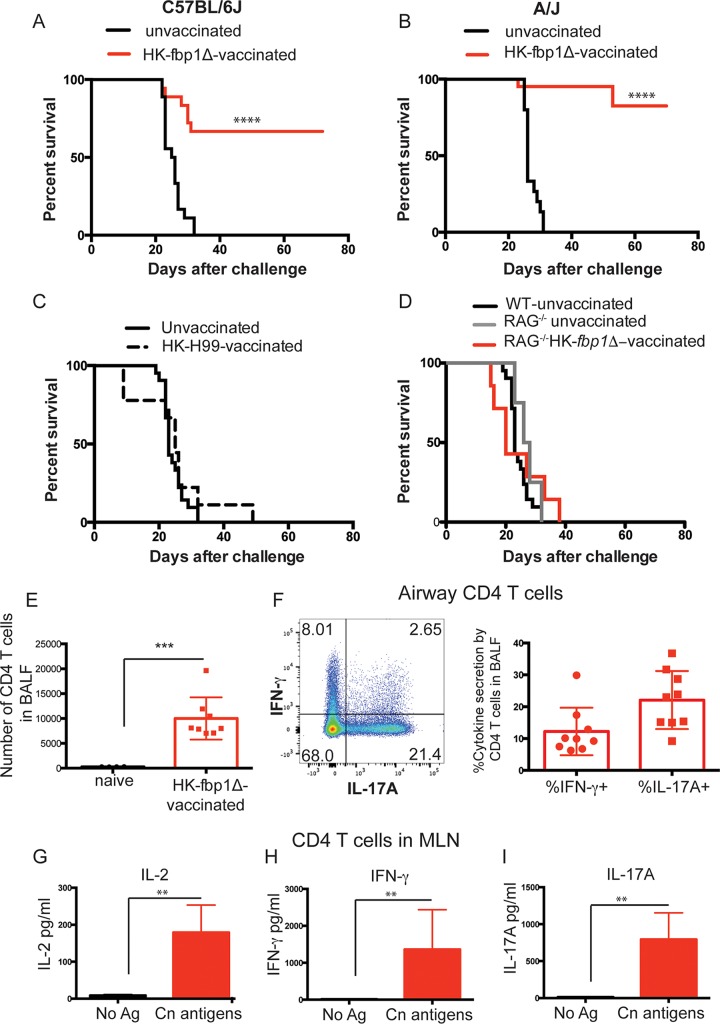
Vaccination with killed *fbp1*Δ mutant cells confers protection against H99 infection. Mice were vaccinated with 5 × 10^7^ HK *fbp1*Δ mutant cells i.n. on days −32 and −7. On day 0, vaccinated mice and unvaccinated controls were infected with 10^4^ virulent H99 cells. All of the data shown are for mice in the C57BL/6J background, except for those in panel B, which are for mice in the A/Jcr background. (A) Percent survival after H99 infection of naive mice (black lines) and mice vaccinated with HK *fbp1*Δ mutant yeast (red lines). The data shown are cumulative from three independent experiments with a total of 18 mice per group on the C57BL/6J background. (B) Percent survival after H99 infection (10^4^) of naive mice (black lines) and mice vaccinated with HK *fbp1*Δ mutant cells (red lines). The data shown are cumulative from three independent experiments with a total of 15 unvaccinated and 21 *fbp1*Δ mutant-vaccinated mice on the A/Jcr background. (C) Percent survival after H99 infection of naive mice (black lines) and mice vaccinated with 1 × 10^8^ HK H99 cells (dashed lines). A total of 10 mice per group were analyzed in two independent experiments. (D) Percent survival after H99 infection of naive mice (black lines) and RAG^−/−^ mice that were unvaccinated (gray line) and RAG^−/−^ mice that were vaccinated with HK *fbp1*Δ mutant cells (red lines). The data shown are for 5 to 10 mice per group and are cumulative from two independent experiments. WT, wild type. (E) Total numbers of CD4^+^ T cells recovered from the BALF of naive mice (black symbols) and HK *fbp1*Δ mutant-vaccinated C57BL/6J mice that survived for 72 days after an H99 challenge (red symbols). Each symbol represents one mouse. (F) Representative FACS plot of intracellular cytokine production by CD4^+^ T cells recovered from the BALF of vaccinated C57BL/6J mice 72 days after an H99 challenge. Percentages of IFN-γ- and IL-17A-producing CD4^+^ T cells in vaccinated mice as analyzed in FACS are shown. Each symbol represents one mouse. The data shown are cumulative from three independent experiments. (G to I) CD4^+^ T cells were isolated from the lung-draining lymph nodes of *fbp1*Δ mutant yeast-vaccinated mice that survived an H99 challenge for 72 days. Cytokine secretion in the presence or absence of *C. neoformans* (Cn) antigens (Ag) was examined by ELISA as described in Materials and Methods. The data shown are cumulative from three independent experiments with four or five mice per group and are depicted as the mean ± the standard error of the mean. ****, *P* ≤ 0.0001; ***, *P* ≤ 0.001; **, *P* ≤ 0.01 (determined by log rank [Mantel-Cox] test [A and B] or Mann-Whitney test [E and G to I]).

We next set out to determine whether the enhanced immune response elicited by infection with the *fbp1*Δ mutant could be harnessed to vaccinate mice against a challenge with virulent parental strain H99. Mice challenged with live or heat-killed (HK) *fbp1*Δ mutant cells displayed equal recruitment of CCR2^+^ Ly6C^+^ monocytes (Fig. S2A and B) and developed comparably robust Th1 and Th17 responses (Fig. S2C and E). We thus immunized mice in accordance with the vaccination strategy employed by Zhai et al. to successfully protect mice against an H99 challenge (28). Briefly, mice were immunized with HK *fbp1*Δ mutant cells on days −32 and −7, and on day 0, they were infected with 10^4^ virulent H99 cells. We tested the protective efficacy of vaccination in both the A/Jcr and C57BL/6J genetic backgrounds. Remarkably, vaccination with HK *fbp1*Δ mutant cells conferred significant protection on C57BL/6J (Fig. 7A) and A/Jcr (Fig. 7B) mice. This is in contrast to the 100% mortality of H99-challenged mice that did not receive the vaccination (Fig. 7A and B). Consistent with previously published work, vaccination with HK H99 was unable to confer protection from infection with live H99 (Fig. 7C) (27, 28, 59, 60). HK *fbp1*Δ mutant-vaccinated mice that survived without symptoms for 72 days contained a significant number of CD4^+^ T cells that remained in their airways (Fig. 7E; Fig. S1B). Moreover, airway CD4^+^ T cells recovered from vaccinated mice rapidly produced IFN-γ and IL-17A upon restimulation (Fig. 7F; Fig. S1C and D). Cryptococcus-specific CD4^+^ T cell responses were also sustained in the MLNs of vaccinated mice (Fig. 7G to I; Fig. S1E and G). These observations suggest that enhanced adaptive immune responses might be responsible for the protection of mice vaccinated with the HK *fbp1*Δ mutant. To test the importance of adaptive immunity in vaccine-mediated protection, we vaccinated RAG^−/−^ mice with HK *fbp1*Δ mutant cells prior to a challenge with virulent H99. We found that vaccination with HK *fbp1*Δ mutant cells could not protect lymphocyte-deficient mice from a challenge with virulent H99 (Fig. 7D), demonstrating that lymphocytes are responsible for vaccine-mediated protection in this model. Altogether, these findings demonstrate that the development of a robust host immune response can overcome the pathogenicity of H99.

10.1128/mBio.01828-17.2 FIG S2  C57BL/6J mice were challenged i.n. with 10^6^ live *fbp1*Δ mutant cells (red symbols in white bars) or 10^7^ HK *fbp1*Δ mutant cells (striped bars). (A and B) Total numbers of monocytes and mo-DCs recovered from the lungs of mice on day 3 after a challenge. (C to E) CD4^+^ T cell responses on day 7 after infection. CD4^+^ T cells were purified from the MLNs of challenged mice and stimulated with APCs in the presence or absence of *C. neoformans* sonicated antigens. The data shown are for cells cultured with *C. neoformans* antigens. No detectable cytokines were present in the absence of antigen stimulation. Cytokine secretion in culture supernatants was examined by ELISA 72 h after culture initiation. Data are cumulative from two independent experiments with three or four mice per group. ns, not significant. Download FIG S2, TIF file, 2.6 MB. Copyright © 2018 Masso-Silva et al. 2018 Masso-Silva et al. This content is distributed under the terms of the Creative Commons Attribution 4.0 International license.

## DISCUSSION

In this study, we identified a novel mechanism of virulence in the highly virulent H99 strain that shapes the immunogenicity of *C. neoformans*. In aggregate, our findings are consistent with the interpretation that the Fbp1-regulated pathway shapes the immunogenicity of *C. neoformans*. We found that infection with the *fbp1*Δ mutant allows the development of a robust immune response that protects the host against lethal fungal meningitis. The activation of strong immunity correlated with enhanced recruitment and activation of CCR2^+^ Ly6C^+^ monocytes. We also found that long-term protection from infection with the *fbp1*Δ mutant requires adaptive immune responses. Protective adaptive immune responses were dominated by CD4^+^ T cells of the Th1 and Th17 lineages and accompanied by diminished Th2 cytokine production. Infection with the *fbp1*Δ mutant also induced increased activation of IFN-γ-secreting CD8^+^ T cells. Remarkably, the strong immune response that developed upon a challenge with the *fbp1*Δ mutant was able to control the infection, even though these yeast cells retained normal, measurable expression of the several major virulence factors tested (30; this study). Our findings thus suggest that Fbp1 controls a novel virulence pathway in *C. neoformans* that is independent of GXM synthesis as well as melanin and chitosan production. Further studies are necessary to examine whether Fbp1 affects the production of other known virulence factors or novel mediators of virulence.

Our study also shows that the enhanced immunogenicity of the *fbp1*Δ mutant can be harnessed as an effective vaccine for protection against a challenge with virulent parental strain H99. A few *C. neoformans* mutants have been found to be able to elicit protection against a challenge with highly virulent H99. The first effective vaccine strain developed in the H99 background was produced by enforcing the expression of murine IFN-γ in yeast cells (strain γH99) (61). Vaccination studies with strain γH99 have provided critical proof-of-principle evidence of the importance of type 1 immunity in protection, as well as for the capacity of the host immune response to overcome virulence mechanisms of H99 given the proper inflammatory conditioning (60–62). Studies with simplified vaccines based on β-glucan particles loaded with cryptococcal antigenic extracts have also been found to induce robust Th1 and Th17 responses that correlate with vaccine-mediated protection (9). But what factors control the proper generation of a protective inflammatory response upon infection with parental strain H99? Recent studies identified Znf2 as an important regulator of the yeast-to-hypha transition and immunogenicity of H99 (28). Overexpression of Znf2 (Znf2^oe^) leads to enhanced hyphal growth and the development of protective Th1 and Th17 responses (28). Importantly, vaccination with Znf2^oe^ conferred 100% protection against an H99 challenge on A/Jcr mice (28). Recent studies have also shown that vaccination with sterylglucosidase 1-deficient *C. neoformans* (*sgl1*Δ) or HK chitosan-deficient cells (*cda1*Δ*2*Δ*3*Δ) can similarly induce protective immunity to a challenge with H99 (27, 29). Thus, changes in cell wall structure and composition can alter the host immune response in a manner that promotes enhanced immunity, and can be exploited for vaccination strategies (27–29).

Our studies have now identified Fbp1 as an additional control point for how H99 interacts with the host immune system. Fbp1 is a subunit of the SCF^Fbp1^ E3 ligase complex of the ubiquitin-proteasome system (UPS) in *C. neoformans* (30, 31). The UPS is crucial for the controlled turnover of proteins in eukaryotic cells, and thus plays an important role in the regulation of diverse cellular activities. As part of the SCF^FBP1^ E3 ligase complex, Fbp1 contributes to the specificity of the UPS degradation process by targeting specific proteins for ubiquitination. It is possible that *C. neoformans* Fbp1, similar to other F-box proteins, targets multiple proteins for degradation. Therefore, the observed phenotype of the *fbp1*Δ mutant could be due to the aggregate function of the altered turnover of multiple proteins. Alternatively, the effect of the *fbp1*Δ mutant on the host immune response could be due to the regulation of just one factor. A thorough analysis of Fbp1 targets has so far failed to identify Znf2 or chitin deacetylases (CDAs) as targets of Fbp1 regulation (data not shown). Thus, Fbp1 may act independently of Znf2 and CDA genes to regulate the immunogenicity of H99. Given that Znf2 and Fbp1 both have multiple as-yet-undefined targets, it could be that there is a convergence of these two immunogenic strains on a common downstream target.

In addition to demonstrating the enhanced immunogenicity and vaccine potential of the *fbp1*Δ mutant, our study indicates that the induction of protective adaptive immunity to this strain correlated with increased recruitment of CCR2^+^ Ly6C^+^ monocytes. Our studies suggest that an Fbp1-regulated target(s) affects host factors for the optimal recruitment and maturation of CCR2^+^ Ly6C^+^ monocytes. The importance of this recruitment is demonstrated by the increased susceptibility of CCR2-depleted mice to *fbp1*Δ mutant yeast infection. CCR2^+^ monocytes are precursors of monocyte-derived macrophages and dendritic cells (mo-DCs) that are important for controlling multiple infections (21, 22, 57, 63). In previous studies, we showed that mo-DCs are crucial for the priming and Th1 differentiation of *Aspergillus fumigatus*-specific CD4^+^ T cell responses (17, 19). Similarly, CCR2^+^ monocytes and their derivative cells are required for the induction of protective immunity to *B. dermatitidis* and other clinically important fungal pathogens (18, 20–22, 56, 57, 63). Importantly, inhibition of CCR2^+^ monocyte recruitment has been shown to be a crucial mechanism of virulence exploited by *B. dermatitidis* (18, 56). Our findings suggest that inhibition of CCR2^+^ monocyte recruitment is similarly an important point of regulation by Fbp1-controlled targets. Inhibition of CCR2^+^ monocyte recruitment might be a common mechanism of virulence exploited by pathogenic fungi. *B. dermatitidis* has been found to produce a serine protease (DppIVA) that actively cleaves host chemokines, thus affecting monocyte recruitment (56). The Fbp1-controlled target is unlikely to be an activate protease, since HK *fbp1*Δ mutant cells were able to induce monocyte recruitment and vaccine-mediated protection. Instead, it might be that changes in cell wall structure allow better recognition by host cells, and the increased production of CCR2-recuiting chemokines. The future identification of Fbp1-regulated factors that target this pathway will broaden our understanding of the mechanism of *C. neoformans* virulence. Our studies also suggest the potential therapeutic benefit of future interventions aimed at increasing host factors that promote CCR2^+^ monocyte recruitment or inhibitors of fungal products as a means to enhance host immunity to cryptococci and other clinically relevant fungal pathogens.

## MATERIALS AND METHODS

### Mice.

Age- and sex-matched mice of the A/Jcr and C57BL/6J genetic backgrounds were obtained from the Jackson Laboratories. CCR2-DTR mice in the C57BL/6J background were generated as previously described (17, 64). In studies with CCR2-DTR mice, sex- and age-matched littermates were used as controls. RAG-1^−/−^ lymphopenic mice were purchased from the Jackson Laboratories. IL-17A-deficient mice were generously provided by the Edelblum laboratory. CCR2-DTR mice were maintained and bred at the Rutgers-New Jersey Medical School Cancer Center Research Animal Facility (CC-RAF) under specific-pathogen-free conditions. Animal studies were performed at the CC-RAF or at the Public Health Research Institute animal facility. All studies were conducted in accordance with biosafety level 2 protocols and procedures approved by the Institutional Animal Care and Use Committee (IACUC) and Institutional Biosafety Committee of Rutgers University.

### *C. neoformans* strains and growth conditions.

*C. neoformans* var. *grubii* (serotype A) H99, the isogenic *fbp1*Δ mutant, and the complemented (*fbp1*Δ *FBP1*) mutant were previously described (30). All strains were streaked onto a yeast extract-peptone-dextrose (YPD) plate, and incubated at 30°C for 2 days. A single colony was inoculated into 2 ml of YPD medium in a 14-ml polypropylene round-bottom tube, and incubated overnight at 30°C with shaking at 250 rpm. The overnight culture was washed three times with 1× phosphate-buffered saline (PBS), and the concentration of yeast cells was determined by counting with a hemocytometer. The inocula were adjusted to concentrations of 10^5^ H99 cells and 10^6^ *fbp1*Δ mutant cells in a 50-μl volume. After vaccination, mice were challenge with 10^4^ H99 cells in a 50-μl volume.

### Capsule production and GXM purification.

To examine capsule production, 5-µl volumes of overnight cultures were inoculated onto minimal medium (MM) and incubated at 30°C for 3 days. Capsule was visualized by India ink negative staining and observed at a magnification of ×100 with an Olympus CX41 microscope equipped with an Infinity digital camera (Olympus, NJ). The total secreted polysaccharides were purified from a 500-ml YPD culture of each strain by the cetyltrimethylammonium bromide precipitation method as previously described (65). The total amount of GXM was determined by the phenol sulfuric acid method. Purified GXM was sent to the Comprehensive Carbohydrate Research Center at the University of Georgia for one-dimensional proton nuclear magnetic resonance (NMR) spectroscopic analysis.

### Measurement of cell surface mannoprotein, chitin, and chitosan levels.

Mannoprotein staining was done by the method of Lev et al. (33). In brief, fungal cells were grown on YPD overnight and resuspended in PBS supplemented with 0.5% gelatin at a final optical density at 600 nm of 0.5 for 30 min. One hundred microliters of each suspension was coincubated with 10 μl of ConA-FITC (2.5-mg/ml stock; Sigma) for 30 min at room temperature. The extent of ConA-FITC binding was determined by flow cytometry.

Cryptococcus chitin and chitosan measurements were done as described by Baker et al. (34).

### Infections, CCR2^+^ depletion, and vaccinations.

All infections were performed by intranasal (i.n.) inoculation. Mice were anesthetized with 100 μl of a ketamine (12.5 mg/ml)-xylazine (1 mg/ml) mixture prior to inoculum instillation into the nostrils. Various inoculum doses ranging from 1 × 10^4^ to 1 × 10^6^ H99 and *fbp1*Δ mutant cells were used as described in the figure legends. For survival experiments, mice were monitored daily for the development of disease symptoms, and euthanized in accordance with IACUC guidelines. For analysis of parameters of host immunity, lungs, bronchoalveolar lavage fluid (BALF), and MLNs were harvested and processed as previously described (46, 47). In brief, BALF was collected in 3 ml of 1× PBS. A catheter was inserted into the trachea of each animal after euthanasia, and airway-infiltrating cells were obtained by lavage with 1 ml of 1× PBS at a time, which was repeated three times. Lungs were perfused with 10 ml of 1× PBS. Single-cell suspensions were prepared by enzymatic digestion with type IV collagenase as previously described (46, 47). Effective depletion of CCR2^+^ cells was achieved by intraperitoneal administration of 250 ng of DT as previously described (57). CCR2-DTR and control littermates were treated with the same dose of DT in accordance with the injection regimen shown in Fig. 6A. For vaccination experiments, the *fbp1*Δ mutant was grown overnight and washed three times in 1× PBS. The concentration of *fbp1*Δ mutant cells was adjusted to 1 × 10^9^/ml, and they were HK by incubation at 75°C for 45 min. Each mouse was immunized by 50 μl of HK cells (5 × 10^7^ cells) and boosted on days −31 and −7 days prior to a challenge with H99. On day 0, mice were each challenged i.n. with 1 × 10^4^ live H99 cells.

### Lung processing.

Single-cell suspensions of pulmonary cells were prepared as previously described (46, 47). In brief, lung tissue was minced in 5 ml of 1× PBS containing 3 mg/ml type IV collagenase (Worthington). Samples were incubated at 37°C for 45 min and washed three times with 1× PBS. After digestion, residual red blood cells (RBCs) were removed with RBC lysis buffer. The total number of lung cells collected from each sample was determined by counting the cells in five squares of a counting chamber with an inverted light microscope at ×40 magnification. Lung cell suspensions were used for RNA extraction and flow cytometry (as described below), as well as for CFU determination by plating serial dilutions.

### Intracellular cytokine staining of T cells harvested in BALF.

BALF was harvested in PBS as described above. All collected cells were pelleted and resuspended in 100 μl of RPMI containing 10% fetal calf serum (FCS), penicillin-streptomycin (2,200 U/ml; Gibco), and a gentamicin sulfate solution (1 mg/ml). BALF cells were then plated in a 96-well round-bottom plate and restimulated with BD Leukocyte Activation Cocktail containing BD GolgiPlug (BD Biosciences). Stimulations were done in accordance with the manufacturer’s instructions. Six hours after activation, BALF cells were surface stained with fluorescently labeled antibodies to Thy1.2, CD4, and CD8. Samples were fixed in 1% paraformaldehyde overnight. Prior to intracellular staining, the samples were permeabilized with 1× BD-Perm/Wash buffer in accordance with the manufacturer’s instructions. Intracellular cytokine staining was done with fluorescently labeled antibodies to IFN-γ, IL-17A, and tumor necrosis factor alpha (TNF-α) diluted in 1× BD-Perm/Wash for 30 min on ice. Samples were immediately washed and analyzed by flow cytometry as described below.

### CD4 T cell recall responses and antibody neutralization.

Lung-draining lymph nodes (MLNs) were collected after euthanasia and placed in 10 ml of 1× PBS. Total lymphocyte cell suspensions were prepared by gently releasing the cells into the PBS by applying pressure to the lymph node with the frosted ends of two glass slides. Repeated pressure was applied until the tissue was reduced to the smallest size possible. Samples were collected and processed in the same way individually. For CD4 T cell isolation, individual samples from each group were pooled (four or five mice). CD4^+^ T cells were purified with a negative-sorting CD4^+^ isolation kit (Miltenyi Biotec, Inc., Auburn, CA). CD4 T cells were isolated in accordance with the manufacturer’s instructions and consistently found to be >90% pure, as assessed by flow cytometry. Purified CD4^+^ T cells (2 × 10^5^) were cultured with 3 × 10^5^ T-cell-depleted antigen-presenting cells (APCs) in RPMI containing 10% FCS, penicillin-streptomycin (2,200 U/ml; Gibco), and a gentamicin sulfate solution (1 mg/ml). The cultures were plated in flat-bottom 96-well plates and incubated at 37°C with 5% CO_2_ for 72 h. APCs were prepared from the spleens of syngeneic, uninfected donor mice. In brief, splenic cell suspensions were depleted of T cells by antibody-complement-mediated lysis. Splenic cells were incubated with anti-Thy1.2 antibodies and rabbit complement (Low Tox; Cedarlane Labs, Hornby, Ontario, Canada) at 37°C for 60 min as previously described (47). To measure *Cryptococcus*-specific responses, CD4-APC cultures were incubated with sonicated H99 yeast cells as a source of fungal antigens. The amount of antigen used was adjusted to a multiplicity of infection of 1:1.5 (APC-to-yeast cell ratio). The fungal growth inhibitor voriconazole was used at a final concentration of 0.5 mg/ml to prevent any fungal cell outgrowth during the culture period. After 72 h after culture initiation, supernatants were collected for cytokine analysis by enzyme-linked immunosorbent assay (ELISA). For IL-2 and TNF-α, ELISA kits from BD-OptEIA were used. IL-4, IL-5, IL-13, IFN-γ, and IL-17A (homodimer) ELISA kits were purchased from EBioscience. For neutralization of IFN-γ, IL-17A^−/−^ mice were injected intraperitoneally with an anti-IFN-γ neutralizing antibody (clone XMG1.2 from BioXCell). Control wild-type mice were injected with an isotype control (clone 2A3 from BioXCell). Both groups of mice were injected 6 h prior to i.n. infection with the *fbp1*Δ mutant. Infected mice were treated with antibodies every other day for 15 days (a total of eight doses per mouse).

### Flow cytometry.

Lung single-cell suspensions were stained for monocytes {CD45 [30-F11 APC-Cy7], CD11b [M1/70-peridinin chlorophyll protein (PerCP)-Cy5.5], Ly6C [AL-21–phycoerythrin (PE)]}, mo-DCs (CD45 [30-F11–APC-Cy7], CD11b [M1/70-PerCP-Cy5.5], Ly6C [AL-21–PE], CD11c [N418-Pacific blue], major histocompatibility complex class II I-A/I-E [M5/11.415.2-Alexa Fluor 700]), neutrophils (CD45 [30-F11–APC–Cy7], CD11b [M1/70-PerCP-Cy5.5], Ly6C [AL-21–PE], and Ly6G [1A8-APC]), CD4 T cells (CD45 [30-F11–APC–Cy7], CD4 [RM4-5–Pacific blue]), CD8 T cells (CD45 [30-F11–APC–Cy7], CD8α [53-6.7–FITC]), and B cells (CD45 [30-F11–APC–Cy7], B220 [RA3-6B2–APC–Cy7]). All antibodies used for lung staining and MLNs were from BD Biosciences. BALFs were subjected to cell surface staining for T cells with Thy1.2 (53-2.1–PE–Cy7) and CD4 (RM4-5–Pacific blue) and intracellular cytokine staining (ICCS) for IFN-γ (XMG1.2-PE), IL-17A (eBio17B7 APC), and TNF-α (MP6-XT22–Alexa Flour 700) in accordance with standard procedures. Most of the antibodies and reagents used for cell surface staining and ICCS were from BD Biosciences, except those used for IL-17A, which were obtained from eBioscience, Inc. All samples were analyzed with a BD LSRII flow cytometer and FlowJo software (Tree Star, Inc.).

### RNA extraction and qRT-PCR.

Total RNA was extracted from lungs with Trizol (Invitrogen). Relative mRNA levels were determined by quantitative reverse transcription (qRT)-PCR. One microgram of total RNA was reverse transcribed with the High Capacity cDNA RT kit (Applied Biosystems). TaqMan Fast Universal PCR master mix (2×) No Amp and TaqMan probes (Applied Biosystems) for each gene were used and normalized to glyceraldehyde-3-phosphate dehydrogenase (GAPDH). Gene expression relative to that of a naive sample was calculated by the ΔΔ*CT* method.

### Statistics.

Statistical analysis of *in vivo* and *in vitro* parameters of antifungal immunity was performed by nonparametric Mann-Whitney test in GraphPad Prism version 7 software. For multiple-comparison analysis of four or more groups, a nonparametric Kruskal-Wallis test was employed by using Prism software. Analysis of data with these nonparametric tests was done to avoid making assumptions about the normal distribution of data points, and because they are considered to be more stringent tests for small samples.

### Ethics statement.

The animal studies described here were compliant with all of the applicable provisions established by the Animal Welfare Act and the Public Health Services Policy on the Humane Care and Use of Laboratory Animals. All studies were performed in accordance with study protocols reviewed and approved by the IACUC of the Rutgers University Newark campus.
