# Supplementary figures and images for: The F-Box Protein Fbp1 Shapes the Immunogenic Potential of Cryptococcus neoformans
Source: mBio. 2018 Jan 9;9(1):e01828-17. doi: 10.1128/mBio.01828-17 (PMC5760740; doi:10.1128/mBio.01828-17)

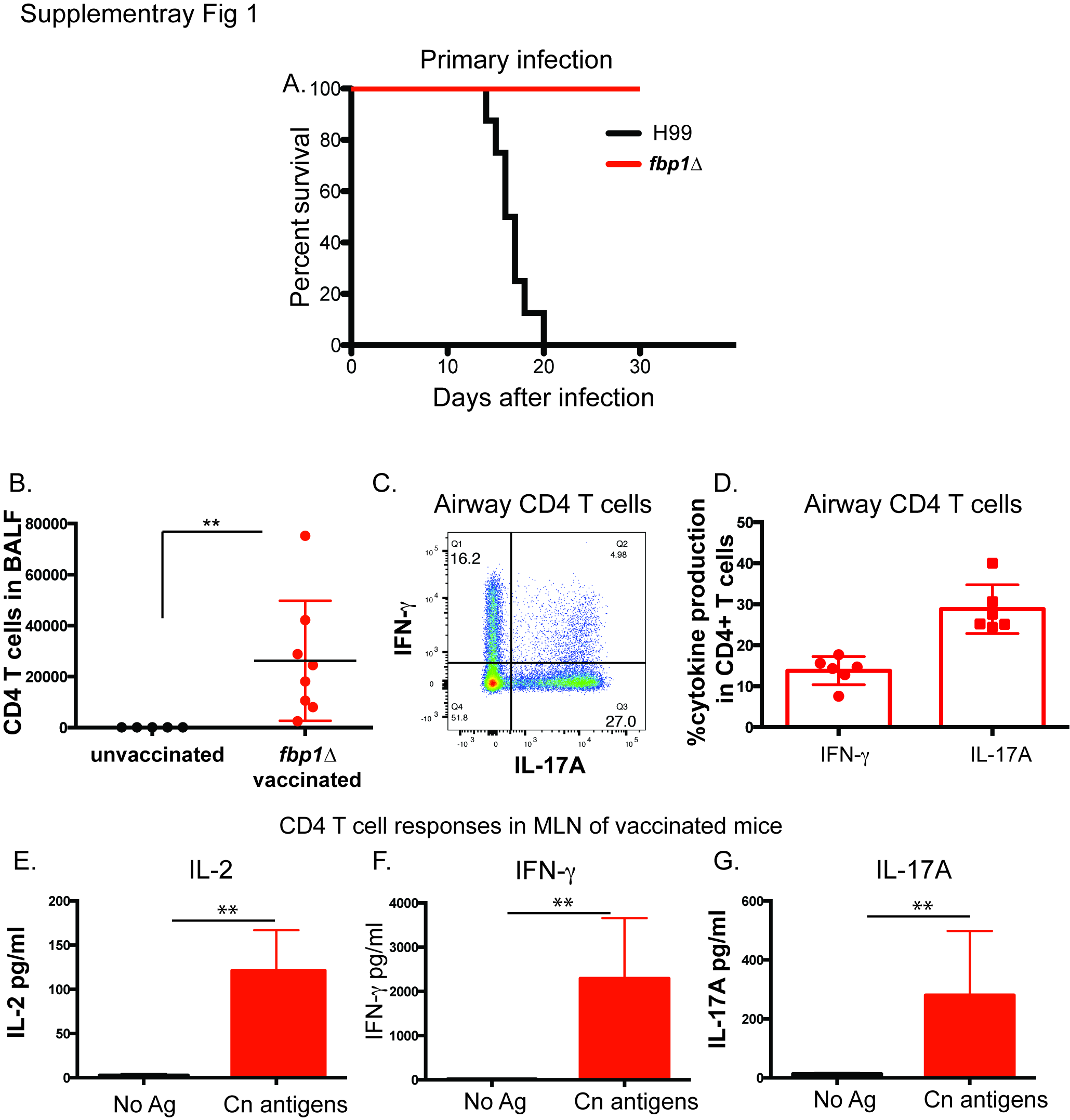

Supplement: FIG S1 [file mbo001183663sf1.tif]

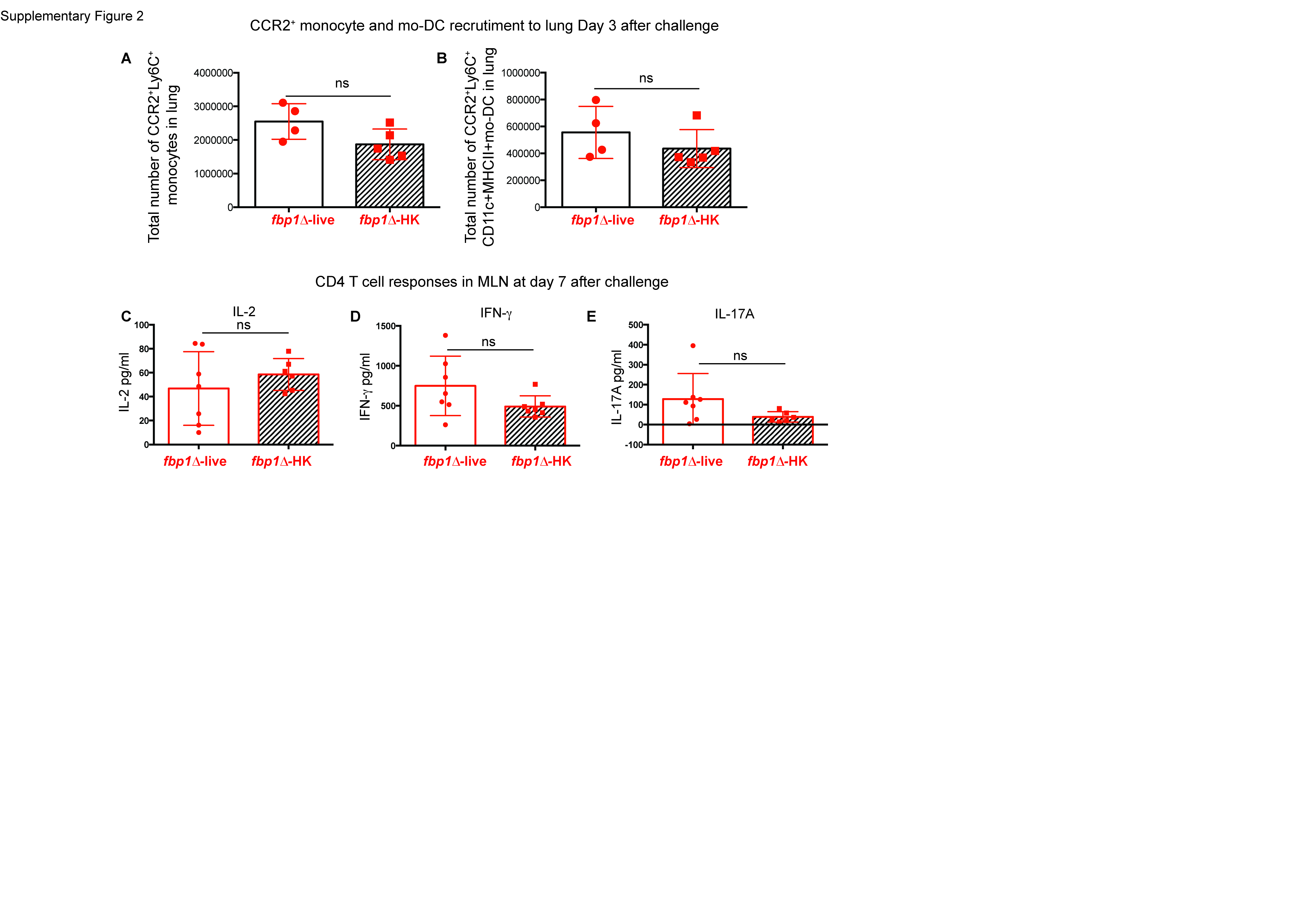

Supplement: FIG S2 [file mbo001183663sf2.tif]
